# A diagnostic phase III/IV seamless design to investigate the diagnostic accuracy and clinical effectiveness using the example of HEDOS and HEDOS II

**DOI:** 10.1177/09622802241227951

**Published:** 2024-02-07

**Authors:** Amra Pepić, Maria Stark, Tim Friede, Annette Kopp-Schneider, Silvia Calderazzo, Maria Reichert, Michael Wolf, Ulrich Wirth, Stefan Schopf, Antonia Zapf

**Affiliations:** 1 Institute of Medical Biometry and Epidemiology, 37734 University Medical Center Hamburg-Eppendorf (UKE), Hamburg, Germany; 2 Department of Medical Statistics, University Medical Center Göttingen, Göttingen, Germany; 3 Division of Biostatistics, German Cancer Research Center (DKFZ), Heidelberg, Germany; 4 CRI—The Clinical Research Institute, Munich, Germany; 5 Clinic for General, Visceral and Transplant Surgery, Hospital of the Ludwig-Maximilians-University, Munich, Germany; 6 RoMed Klinik Bad Aibling, Academic University Hospital of the Technical University of Munich, Bad Aibling, Germany

**Keywords:** Seamless design, diagnostic accuracy, adaptive design, external controls, power prior

## Abstract

The development process of medical devices can be streamlined by combining different study phases. Here, for a diagnostic medical device, we present the combination of confirmation of diagnostic accuracy (phase III) and evaluation of clinical effectiveness regarding patient-relevant endpoints (phase IV) using a seamless design. This approach is used in the Thyroid HEmorrhage DetectOr Study (HEDOS & HEDOS II) investigating a post-operative hemorrhage detector named ISAR-M THYRO® in patients after thyroid surgery. Data from the phase III trial are reused as external controls in the control group of the phase IV trial. An unblinded interim analysis is planned between the two study stages which includes a recalculation of the sample size for the phase IV part after completion of the first stage of the seamless design. The study concept presented here is the first seamless design proposed in the field of diagnostic studies. Hence, the aim of this work is to emphasize the statistical methodology as well as feasibility of the proposed design in relation to the planning and implementation of the seamless design. Seamless designs can accelerate the overall trial duration and increase its efficiency in terms of sample size and recruitment. However, careful planning addressing numerous methodological and procedural challenges is necessary for successful implementation as well as agreement with regulatory bodies.

## 1 Introduction

The assessment of a diagnostic test is part of a process containing several phases^1–3^ involving the evaluation in terms of technical efficacy and feasibility, diagnostic accuracy and clinical effectiveness. Each phase is in general conducted as separate studies with their own objectives, planned individually and performed sequentially with often essential time intervals in between as well as different sample sizes and follow-up times. One approach to more efficient studies in terms of sample size and follow-up times is the use of a seamless design.

In therapeutic research, several studies have already been conducted according to the principle of seamless designs or are currently in the implementation phase.^4–7^ The potential of seamless designs in diagnostic research has been discussed by Vach et al.,^3^ but to our knowledge they have not been implemented so far. Considering two consecutive study stages, Vach et al.^3^ distinguish three types of seamless designs, which essentially differ in terms of how much data is integrated from the first stage into the second. In the first type, no data is shared, but the logistics. In the second type, data from the first stage is re-used in the second stage. The last type describes the two stages running in parallel (or with some overlap), where collected data is used in both stage-specific analyses.^3^

In this article, we focus on the seamless transition between a diagnostic phase III study (confirmatory diagnostic accuracy trial) and a diagnostic phase IV study (randomized test-treatment trial with a patient-relevant endpoint). Diagnostic phase III accuracy studies are performed to assess how well a diagnostic test can distinguish between patients with and without a target condition by primarily evaluating the diagnostic accuracy, that is, sensitivity and specificity of the investigating diagnostic procedure.^2,8^ In randomized test-treatment studies the primary objective is the assessment of the clinical effectiveness of a diagnostic procedure in terms of clinically relevant patient outcomes.^9–12^ To the best of our knowledge, no study has been planned or conducted applying a seamless design to evaluate a diagnostic procedure with respect to its accuracy as well as clinical effectiveness.

We describe the seamless design using the Thyroid HEmorrhage DetectOr Study (HEDOS) as a case study. The aim of this trial is to assess the diagnostic accuracy based on a pre-defined cut-off value in patients with unknown disease status (stage 1) and subsequently the clinical effectiveness (stage 2) of the medical device ISAR-M THYRO® (investigational product; IP) in detecting clinically relevant hemorrhage following thyroid surgery, compared to standard of care (SOC) monitoring procedures. Data collected in the first stage of the study program will be re-used in the SOC monitoring arm (control group) of the second stage study. This type of seamless design corresponds to Type B of the presented types by Vach et al.^3^ More exactly, the study in stage 2 benefits from the additional controls who participated in stage 1 study of the seamless design.

Viele et al.^13^ presented a spectrum of methods for borrowing external information which range from simply pooling the external data with current trial data to more complex Bayesian methods considering power priors and hierarchical modeling. The power prior assigns a “weight” to the external data. Hence, the external data is incorporated to a certain degree into the current analysis.^14,15^ In a hierarchical model, a distribution across the current and external controls with an explicit coefficient measuring the variation across the data is assumed. Initially, assumptions regarding the prior distribution of the coefficient of variation are met which are updated based on the current data. Pocock^16^ assumes in his work that the estimator based on the external data is biased to some unknown degree. A Bayesian prior distribution is assumed for the unknown bias which represents the level of reliance one has in the external data. The point estimator of the control group (randomized and additive controls) will be combined using a weighted sum of sample estimates in order to down-weight the data from the additive controls. Another option would be the “test and pool” strategy where the pooling of the external and current data is carried out if a significance test on the equality of population estimators results in a significance level greater than some pre-specified value.^13^ More details about the analysis including external data can be found in Section 3.4.2.

Between the stages of the seamless design, an unblinded interim analysis is carried out to estimate the diagnostic accuracy of the IP and the incidence of alerts detected in the IP arm and the control arm. Based on these estimates, the required sample size for the second stage study is derived. In addition, a blinded recalculation of the sample size based on prevalence is planned as part of the diagnostic accuracy study (stage 1) to account for the uncertainty assumptions regarding prevalence in the planning phase of the study.

The scope of this article is the methodological review of the seamless design applied in HEDOS. We focus on the statistical as well as practical and regulatory issues of the implementation, specifically the characteristics of a seamless design integrating two different diagnostic trial phases.

The paper is structured as follows: firstly, the HEDOS study is presented including all key features of the study design. Subsequently, all statistical issues considered in the study are demonstrated. Finally, the study rationale and implementation challenges as well as regulatory aspects are discussed.

## 2 Case study presenting a diagnostic phase III/IV seamless design

The study design of HEDOS is planned as a seamless design of Type B in which a confirmatory phase III diagnostic accuracy study (stage 1, HEDOS) and a proceeding diagnostic phase IV randomized test-treatment study (stage 2, HEDOS II) are integrated with each other (phase labels according to Koebberling et al.^1^). Hence, this seamless design combines two stages in one overall study concept.

### 2.1 Background

After thyroid surgery, 0.6–4% of patients develop post-operative hemorrhage.^17^ Only 40% of these complications occur within 8 h after of surgery while 90% of post-operative hemorrhage occurs within the first 48 h.^18^ In most cases, these are rapidly progressing complications that require immediate intervention, as the hemorrhage is caused by arterial hemorrhage sources^17–20^ as we could previously proof the correlation of increased cervical pressure and decrease in cerebral perfusion and cerebral oxygenation in an animal experimental series.^21^ Up to 0.6% of patients with post-operative hemorrhage die, while others suffer from hypoxic brain damage.^19^ The cause of death or of hypoxic brain damage in these patients is a lack of oxygen assumed to be caused by a cervical compartment syndrome. Thyroid surgery patients are mostly female (75%) and young. This makes serious complications such as post-operative hemorrhage with its risk for additional morbidity and life-threatening consequences particularly tragic.

In a clinical study with post-operative pressure measurements^22^ it could be demonstrated that post-operative hemorrhage leads to a continuous increase in pressure in the neck without interruption as it is observed when coughing and pressing. Systematic invasive pressure measurement in the thyroid compartment after surgery might detect a continuous increase in pressure which is often caused by a growing hematoma, indicating serious post-operative hemorrhage at a much earlier time compared to state-of-the-art diagnostic workflow. In routine clinical care, detection of serious hemorrhage depends on the patients alerting symptoms even if post-operative intermittent monitoring of vital parameters and wound conditions is performed according to current medical guidelines^23^ and local instructions. Device-based, continuous hemorrhage detection within 36–48 h after surgery would allow to objectively measure an increase in cervical pressure before symptoms occur. The IP cannot prevent bleeding complications, but early detection is important to avoid additional morbidity and potential mortality and thus to optimize the outcome. Thus, the risk of serious complications like hypoxic brain damage and death caused by post-operative hemorrhage is minimized. In addition, the intervention team would be able to fine-tune necessary actions during the rescue procedure based on objective pressure values, for example, the decision to open cutaneous sutures immediately or later in the operation theater, and therefore reduce additional perioperative morbidity and increase patients’ safety.

### 2.2 Definition of key terms

The following terms are used in the remaining part of the paper and are defined, unless otherwise stated, as follows:

In the study, the reference standard test is defined as SOC monitoring which is assumed to be imperfect. Patients are monitored according to routine clinical care. Based on clinical signs, a decision is made whether a revision surgery is necessary or not.

The investigational medical device product (IP) used within this study is ISAR-M THYRO®, a diagnostic device of class IIb according to EU regulation 2017/745 for early detection of hemorrhage following thyroid surgery. IP-based alerts triggering revision surgery are defined by IP pressure measures which exceed a predefined cut-off. By objectively measuring the cervical pressure, it is possible to assess whether or not there is a need for intervention.

A true-diseased patient is characterized by a post-operative revision surgery which confirmed post-operative hemorrhage needing intervention (clinically relevant hemorrhage). In routine clinical care, symptomatic hemorrhage will in any case be noticed at some point and a revision surgery will confirm if a relevant hemorrhage was present. Thus, the gold standard defining the true disease state of a patient is considered to be the occurrence of a post-operative hemorrhage with need for revision surgery as detected by routine clinical care monitoring.

Data arising from stage 1 and being included in stage 2 are referred to as external, additional or historical data throughout the manuscript.

In stage 1, a nuisance parameter will be estimated based on interim results of the ongoing study in order to adjust the initially planned sample size. This approach refers to a study design with an internal pilot study.^24^

### 2.3 Study design and objectives

HEDOS is a prospective, international, multi-center, diagnostic phase III/IV seamless design aiming to evaluate the diagnostic accuracy and clinical effectiveness of the IP ISAR-M THYRO in patients after thyroid surgery. Two different study types are combined in one single study with two stages:

1. A prospective, single-arm, international, multi-center, blinded, observational, diagnostic accuracy study with the IP (diagnostic phase III study, HEDOS).
2. A prospective, two-arm, international, multi-center cluster-randomized test-treatment study comparing the diagnostic IP to a control group regarding patient outcome (diagnostic phase IV study, HEDOS II).

In this seamless design, the confirmatory diagnostic phase III study refers to stage 1, whereas the proceeding diagnostic phase IV study refers to stage 2. The diagnostic procedure under investigation is the use of the IP which is designed to detect clinically relevant cervical hemorrhage within 48 h after the end of index thyroid surgery by continuous measurement of pressure in the former thyroid compartment. In this stage, the IP is compared to the gold standard which indicates whether or not post-operative hemorrhage requiring revision surgery has occurred as detected in routine clinical care. If an IP-based alert is raised, this would require revision surgery. However, in the first stage, the IP is silenced so that no alert is indicated and only routine clinical care procedures will determine the need of revision surgery and subsequent treatment decisions.

A blinded sample size re-calculation based on the incidence proportion of hemorrhage events measured by the gold standard procedure within stage 1 study is planned. After completion of stage 1 of the seamless design, in particular, after market approval of the IP, an unblinded interim analysis is carried out by estimating the diagnostic accuracy of the IP and the incidence of alerts in both arms—post-operative hemorrhage with need for revision surgery as detected in routine clinical care versus silent IP-based alerts. Thereafter, based on this incidence resulting from the gold standard a sample-size re-calculation is performed in order to derive the required sample size of the second stage. At this point, recruitment to the study is temporarily halted to wait for CE-certification of the medical device.

**Figure 1.**
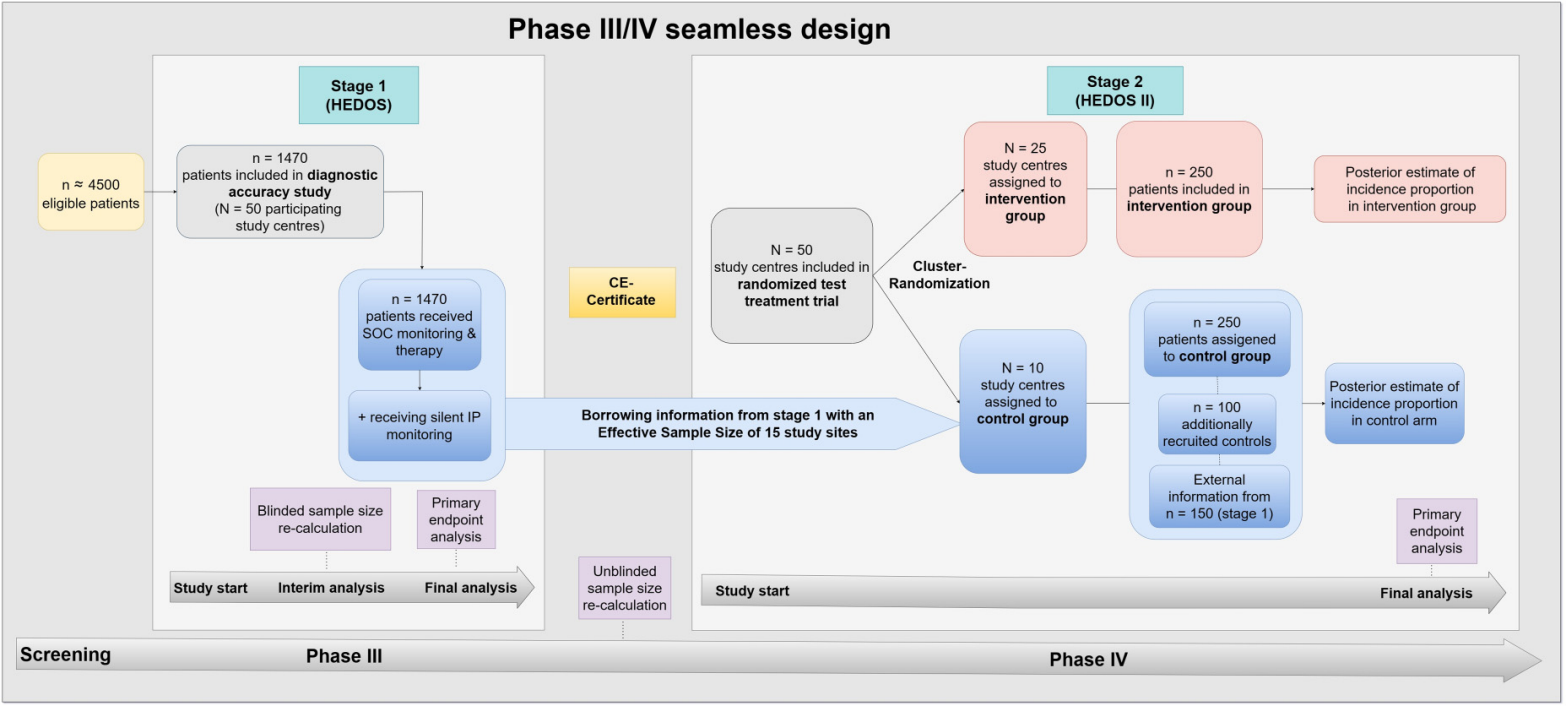
The confirmatory diagnostic accuracy phase III study (HEDOS) and the cluster-randomized test-treatment study (HEDOS II) are connected via the seamless design. The sample sizes in stage 2 are exemplary and serve to illustrate the design. The final numbers are calculated in the interim analysis. N: number of study sites; n: number of patients; SOC: standard of care; IP: investigational product.

The phase IV trial (stage 2) is planned as a cluster-randomized test-treatment trial comparing the effectiveness of the IP versus SOC monitoring procedure in routine clinical care. Cluster randomization will be performed at level of study sites (= hospitals) as it is considered more convenient for clinicians to use the medical device in all participating patients and not switch to the SOC monitoring in between. In the first step, all study sites will be randomized to either apply the IP (intervention group) on patients undergoing thyroid surgery or to perform routine clinical care monitoring after thyroid surgery (control group). Hereby, the control group information is augmented with information coming from the phase III trial in stage 1.^13^ Since the patients in these study sites are monitored and treated according to routine clinical care without any diagnostic or therapeutic intervention by study protocol, we do not expect strong heterogeneity with the control arm of stage 2 study. Thus, the focus of study site recruitment in stage 2 will mainly be on recruiting study sites into the intervention group. Patients belonging to the intervention group will be treated based on the displayed three alert signals by the IP (see Section 3.3.1). The routine clinical care monitoring in the control group indicates if relevant hemorrhage is assumed to be present and more intensive monitoring or further surgery is required just based on clinical symptoms. Figure 1 shows the seamless design. In the following, we will go into detail about the two individual stages.

### 2.4 Stage 1

#### 2.4.1 Objective

The primary study objective of the phase III diagnostic accuracy study is to evaluate the diagnostic accuracy, that is, sensitivity and specificity, of the IP in detecting clinically relevant hemorrhage within 48 h following thyroid surgery using 12 mmHg pressure as cut-off.

In standard clinical care, it is determined by routine clinical monitoring whether a truly diseased patient who has post-operative hemorrhage requiring intervention is identified. Thus, clinically relevant symptomatic hemorrhage will in any case be noticed at some point and revision surgery will determine if a relevant hemorrhage was present. This means that verification bias can be ruled out with a high degree of certainty.

**Figure 2.**
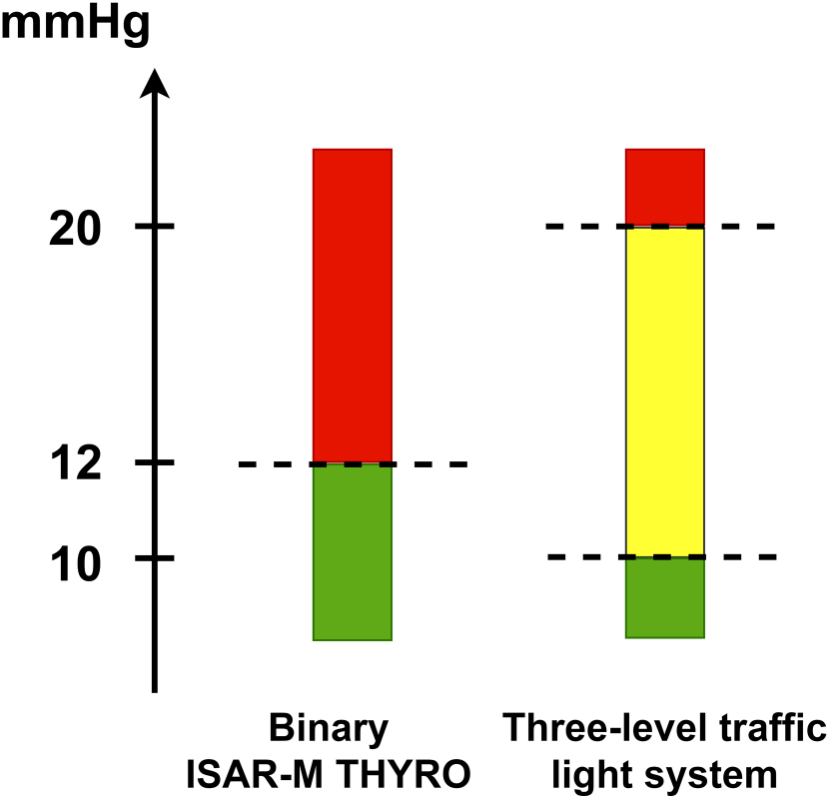
Cut-off values of the binary and three-level traffic light system.

Furthermore, a three-level traffic light decision system will be established and validated in order to assess the corresponding diagnostic accuracy (see Figure 2). Here, the IP will detect three levels with different needs for action: in case of level green (pressure ≤ 10mmHg), no alert is set, so in clinical practice, the patient would be treated as usual. At level yellow (pressure > 10 mmHg to ≤ 20 mmHg), a critical pressure level is reached. Intensified monitoring of the patient would be necessary and staff at the ward and at the operating theater would be alerted without performing surgery yet. At the red level (pressure > 20 mmHg), a post-operative hemorrhage requiring immediate surgery would be indicated. The validated three-level traffic light decision system and the definition of its corresponding subsequent management strategies will be necessary for the second stage of the study program since here the clinical effectiveness of the detector is evaluated using the traffic light system. In particular, the use of an additional area (“yellow warning level”) can help to avoid unnecessary revision surgery, and the need for revision surgery can be identified earlier than with SOC.

**Figure 3.**
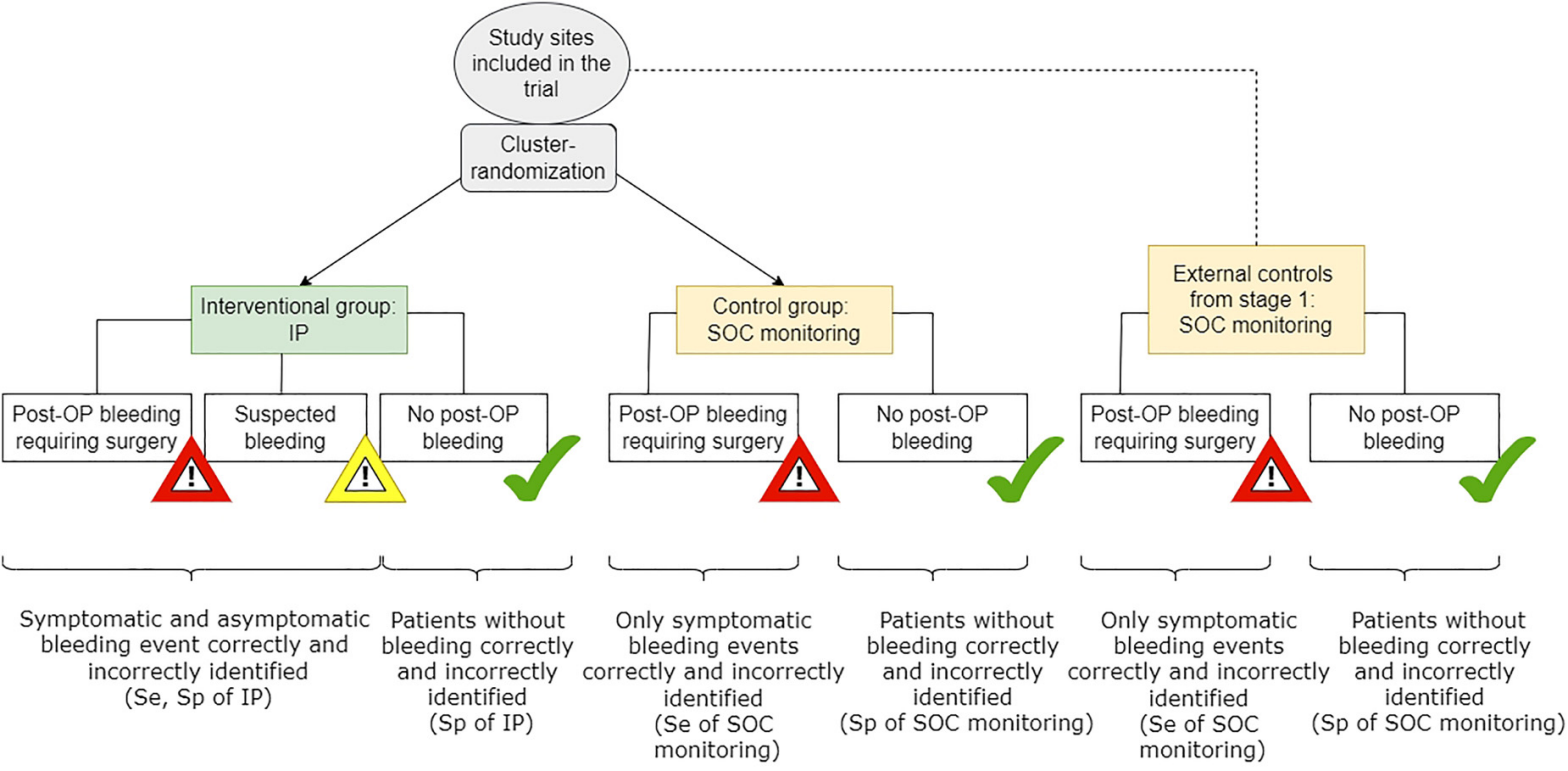
Phase IV randomized-test-treatment study (stage 2). Se: sensitivity; Sp: specificity; IP: investigational product; SOC: standard of care; OP: operation.

However, in routine clinical practice, the IP-based alerts are silenced during stage 1, but the pressure measures are recorded for the statistical analysis. Subsequent treatment decisions are based on the routine clinical monitoring.

The key secondary objective is to assess the safety of the use of IP. Primary and secondary objectives are investigated in a patient cohort representing the routine clinical care population to avoid spectrum bias.

#### 2.4.2 Outcome measures

On patient level, true positive, true negative, false positive and false negative test results represent endpoints in which the gold standard defines the true disease status. On population level, the primary endpoints are defined in the following way:

1. Sensitivity and specificity (co-primary) of the IP for detection of clinically relevant hemorrhage within 48 h following thyroid surgery using 12 mmHg of pressure as cut-off compared to detection in routine clinical care.
2. Area under the Receiver Operating Characteristic (ROC) curve (AUC) for detection of clinically relevant hemorrhage within 48 h following thyroid surgery by the IP in a three-level decision system using 10 and 20 mmHg as cut-offs by calculating the AUC from the resulting pairs of sensitivity and specificity compared to detection in routine clinical care.

All specified cut-offs were determined by clinicians based on case reports and animal experiments.

Secondary endpoint parameters are defined on population level as follows:

- Safety of the use of the diagnostic IP by means of adverse events within 1 month following thyroid surgery
- Sensitivity and specificity of the IP for detection of clinically relevant hemorrhage within 48 h following thyroid surgery in a three-level decision system using 10 and 20 mmHg of pressure as cut-offs compared to detection in routine clinical care
- Positive and negative predictive values of the IP for detection of clinically relevant hemorrhage within 48 h following thyroid surgery using the binary and the three-level decision system compared to detection in routine clinical care.
- AUC for detection of clinically relevant hemorrhage within 48 h following thyroid surgery by the IP in a three-level decision system using varying cut-offs in the range of measurements of the IP compared to detection in routine clinical care.

Clinically relevant hemorrhage and serious adverse events will be adjudicated by an independent Endpoint Review Committee.

Parameters relevant for endpoints within stage 2 study are collected within stage 1 study, too, for example, clinical symptoms triggering surgical intervention of a suspected post-operative hemorrhage detected in routine clinical care, time to clinical or technical alerts, surgical interventions which did not show post-operative hemorrhage needing intervention.

#### 2.4.3 Hypotheses

There are two co-primary hypotheses and one further (third) primary hypothesis. These are tested hierarchically, that is, the third primary hypothesis will only be evaluated in a confirmatory way if both of the co-primary hypotheses yield significant test results.

The first primary hypotheses aim to demonstrate a sufficient sensitivity and specificity of the IP by comparing them to a minimum sensitivity of 80% and a minimum specificity of 65%. The aim of the third primary hypothesis is to demonstrate a sufficient diagnostic accuracy of the three-level traffic light decision system of the IP. In this context, the AUC is calculated for the three-level ordinal outcome of the traffic light system. To test the third primary hypothesis, AUC is compared against a pre-defined minimum AUC of 0.7.

### 2.5 Stage 2

#### 2.5.1 Objective

The primary objective of the phase IV randomized test-treatment study is to demonstrate the clinical benefit of the IP compared to the application of the current SOC detection procedure in routine clinical care in patients after thyroid surgery. Due to the existing risk of post-operative hemorrhage requiring revision surgery after index thyroid surgery, the aim is to investigate whether the use of the IP can detect signals of clinically relevant post-operative hemorrhage earlier and more effectively. Thus, post-operative hemorrhage can be treated in time and undesired hemorrhage-related morbidity for the patient can be minimized.

#### 2.5.2 Outcome measures

Similarly, to the first stage, two primary endpoints are structured in a hierarchical way:

1. The occurrence of a first alerting event, including clinical alerts based on typical clinical symptoms related to potential relevant hemorrhage triggering surgical intervention, for example, vomiting, shortness of breathing, dysphagia, dizziness and technical alerts by the IP is measured on patient level (see Figure 3). The alerting events are then verified by the revision surgery showing that the alerting event indeed needs clinical treatment. Then, on population level, the incidence proportion of the verified alerting events is calculated and analyzed as first primary endpoint.
2. The second primary endpoint is a time-to composite endpoint, measured on patient level and includes all adverse events from start of the index surgery until one month of follow-up. On population level, the corresponding event rates at one month are calculated.

All secondary endpoint parameters are measured on patient level and defined as:

- Death of any cause measured as total mortality.
- Number of hospital nights after end of index thyroid surgery to one month of follow-up.
- Number of surgical interventions for suspected hemorrhage which did not show post-operative hemorrhage needing intervention.
- Length of hospital stay for index thyroid surgery.
- Quality of life measured by EQ-5D questionnaire before index thyroid surgery and one month after.
- Anxiety disorders are measured by the GAD-7 questionnaire before index thyroid surgery and one month after.
- Time to an alerting event in patients with a revision surgery performed which confirmed post-operative hemorrhage needing intervention.

Number of surgical interventions for suspected hemorrhage which did not show post-operative hemorrhage needing intervention

#### 2.5.3 Hypotheses

There are two primary hypotheses which are tested hierarchically. These hypotheses aim to tests for

1. Superiority regarding the verified incidence proportion of alerting events in the intervention group compared to the control group.
2. Non-inferiority regarding the composite endpoint of death of any cause, surgical interventions for suspected hemorrhage which did not show serious bleedings and serious adverse events after index surgery until one month of follow-up in the intervention group compared to the control group. The non-inferiority margin is set to a hazard ratio of 1.2 which is assumed to be clinically relevant set by the clinicians.

## 3 Statistical aspects

### 3.1 Randomization

Only the second stage of the seamless design involves a randomization procedure. This is performed at the cluster level based on participating study sites, which are randomized into either the intervention or control group. Patients are included in the trial at the time of admission to the hospital before their thyroid surgery. Before their surgery patient baseline characteristics are measured. Since information from additional control study sites from stage 1 will be integrated into stage 2 analysis, the ratio of randomization is adjusted to balance the treatment arms in effective sample size (ESS), as described below. The randomization ratio depends on the number of study sites in the control group which still have to be randomized in relation to the study sites in the intervention group. This will be determined during the interim analysis after the end of stage 1. Hence, in the first step, the required number of study sites of stage 2, $N_{2}$ , is re-calculated based on the results of stage 1. Afterwards, the number of control study sites randomized is determined by Nc=N2/N222−ESS1, where the prior ESS borrowed from stage 1, $ESS_{1}$ , represents stage 2 prior informativeness in terms of number of stage 1 study centers, $N_{1},$ available for information borrowing.^25,26^ As a large number of stage 1 study centers is expected compared to stage 2, this value is capped at 15, so $ESS_{1}=$ min( $N_{1},15)$ , which corresponds to 30% of the expected number of study sites included in stage 1 ( $N_{1}\approx 50$ ). Stage 2 prior distribution for the control incidence proportion is robustified in order to adapt to potential conflict between stage 1 and 2 parameters’ estimates, as described in Section 3.4.2. If the assumption of homogeneity between the parameters of the two stages is satisfied, the effective informativeness of the prior will be close to $ESS_{1}.$ The number randomized study sites in the intervention group is Ne=N2/N222. Thus, the randomization ratio on study site level is set at $N_{e}:N_{c}$ . The total number of patients included in the trial is determined by the assumed cluster size and number of clusters.

### 3.2 Sample size

In the planning phase of the study programme, the initial sample size for both stages of the study programme is calculated separately and based on assumptions informed by the literature.

#### 3.2.1 Stage 1

In the first stage of the study programme, sample size calculation is performed for the primary hypothesis which states the superiority considering sensitivity and specificity of the IP against pre-defined minimum thresholds.^27^ For this, we applied the optimal sample size calculation for a single-test confirmatory diagnostic accuracy study.^8^

Based on preliminary data, the sensitivity of the IP is assumed to be 99%, the specificity is assumed to be 95%. The pre-defined minimum sensitivity equals 80%, the minimum specificity is 65%. The assumed prevalence of clinically relevant hemorrhage within 48 h following thyroid surgery is 1.5%.^28^ Because of this low prevalence, the two-sided significance level is set to 5% for sensitivity and specificity, respectively. An overall power of 80% is desired. This leads to a total sample size of 1 470 patients enrolled. Further details on the sample size procedure and formulae are provided in Zapf and Stark.^8^ No drop-outs are assumed, since the outcomes are observed while being in hospital. If the prevalence differs, at least 22 patients with clinically relevant hemorrhage within 48 h following thyroid surgery need to be observed.

Furthermore, for stage 1, an adaptive design is planned to re-estimate the sample size based on the estimated prevalence during a blinded interim analysis. The interim analysis is performed after 50% enrolled and measured study patients of stage 1 study. If the re-estimated sample size is larger than the number of patients recruited at time of interim analysis, recruitment continues until the re-calculated sample size is reached. Otherwise, recruitment stops and the final analysis in stage 1 can be performed. The maximum sample size for stage 1 is set to 4500 patients enrolled and measured. Due to the blinded character of the adaptive design, no adjustment of the type I error rate is necessary.^8,29^ In total, it is expected that 50 study sites will be included in stage 1 study.

#### 3.2.2 Stage 2

The advantage of the IP compared to the standard procedure is the identification of all possible hemorrhages, that is, symptomatic as well as asymptomatic hemorrhages leading to a technical and/or clinical alert (corresponding to the yellow and red level of the three-level traffic light decision system, respectively) (Figure 2). Accordingly, sample size calculation is performed for the first primary endpoint “incidence proportion of alerting events” in the intervention group (technical and/or clinical alerts, corresponding to the red and yellow level) compared to the control group (only clinical alerts, corresponding to the red level) and is based on an independent two-sample proportion test with the Pearson's chi-squared test for cluster-randomized trials with two-sided 5% significance level assuming that the variances are unpooled and that a continuity correction was not used. A formula for the respective sample size calculation is included in the Supplemental Material. The sample size calculation was carried out using nQuery 8.7.2.0.

For initial sample size calculation in stage 2, incidence proportion of alerting events in the two groups base on several parameters:

1. Sensitivity and specificity to ensure that alerts regarding a clinically relevant hemorrhage are correct with a sufficient degree of certainty (derived from stage 1)
2. The incidence proportion of technical and/or clinical alerts overall.
3. The expected incidence proportion of alerts in the different subgroups, that is, in the group where post-operative clinically relevant hemorrhage is correctly as well as incorrectly diagnosed.

Initially, we calculate the sample size needed to achieve a difference in the number of all possible clinically relevant hemorrhages in the red and yellow levels of the IP group compared to the number of symptomatic events in the control group.

Assumptions regarding the diagnostic accuracy of the IP will be estimated from stage 1. So the initial assumptions of a sensitivity of 99% and a specificity of 95% can be adjusted after the unblinded interim analysis.

On the one side, a total of 1.5% of patients after thyroid surgery are expected to have clinically relevant hemorrhage events, which also applies in the previous study phase. Therefore, only this proportion of events might be detected in the control group at most. On the other hand, it is assumed that in the control group, most patients without symptoms in fact do not have any clinically relevant hemorrhage. Hence, the specificity of the SOC monitoring procedure is expected to be 99%. Based on the prevalence of asymptomatic and symptomatic events the IP might detect additionally in the yellow area (asymptotic hemorrhage) and on the defined specificity of the gold standard method, we can calculate how high the sensitivity in the control group could be. The higher the prevalence of symptomatic and asymptomatic events, the less likely it is that the gold standard method will detect them. In the Supplemental material of the paper, a more detailed description of this calculation and different scenarios are presented.

Applying these assumptions, the calculation of the expected incidence proportion in both study arms follows Hot et al.^9^ and results in 2.4% for the control arm and 11.5% in the IP arm. It is expected that 50 study centers will be included in stage 2 trial with the in Section 3.1 derived randomization ratio. Assuming an intraclass correlation coefficient of 0.05 and a cluster number of 25 per study group, a minimum cluster size of 10 patients must be achieved to detect an odds ratio of 5.22 with a power of 90%. Thus, at least 250 patients per group and 500 patients in total are required in stage 2. For the control group, including the additional controls from stage 1, this leads to $N_{c}=25-ESS_{1}=10\;$ study sites, who still need to be randomized. With the resulting randomization ratio of 1:2.5, this corresponds to 10 centers randomized to the control group and 25 randomized to the intervention group each with a minimum of 10 patients on average in each study site. A possible dropout rate will be estimated after completion of stage 1 and will be incorporated in the final sample size calculation of stage 2.

### 3.3 Interim analyses

The first interim analysis is planned during stage 1 study. In particular, a blinded interim analysis to re-calculate the sample size is performed after 50% enrolled and measured study patients of stage 1 study. Using the optimal sample size calculation by Stark and Zapf,^8^ the sample size will be re-calculated based on the estimated prevalence. If the re-calculated sample size is larger than the size of the internal pilot study, recruitment continues until the re-calculated sample size is reached. Otherwise, recruitment stops and the final analysis can be performed. The maximum sample size is set to 4500 patients enrolled and measured. Due to the blinded character of the adaptive design in which the sensitivity and specificity of the IP are not revealed at this time, no adjustment of the type I error rate is necessary.^8,29^

A second pre-specified unblinded interim analysis is planned after completion of stage 1. The aim of the interim analysis is to estimate the incidence proportion of hemorrhages based on the gold standard, that is, the SOC monitoring, as well as sensitivity and specificity of the IP and to re-calculate the sample size based on these results. Since the SOC monitoring is performed without knowledge of the IP results (blinding), and the alerts in the detector are set completely silent, the estimators should be unbiased. Early stopping for efficacy is not possible because only the results of SOC monitoring from the first stage are included in stage 2, since the treatment of patients in stage 1 is based exclusively on these results. Since there is no unblinding of the group assignments of stage 2 in this interim analysis, the type 1 error does not need to be adjusted for the final analysis of stage 2.

In addition, the drop-out rate can also be estimated in the interim analysis of stage 1 and taken into account in the sample size re-calculation.

In case of futility of stage 1 study, stage 2 study would not be continued. This can only be done if the IP achieves its CE-certification, which is linked to the success of stage 1 study.

### 3.4 Statistical analysis

#### 3.4.1 Stage 1

In the first pair of primary hypotheses, sensitivity and specificity are considered as co-primary endpoints. They are combined via the Intersection-Union Test. The global null hypothesis can only be rejected if both individual null hypotheses of sensitivity and specificity are rejected. Hence, the global type I error does not need to be adjusted^30^ The corresponding global alternative hypothesis states that the IP exceeds a pre-defined minimum sensitivity of 80% and specificity of 65%. These numbers are based on clinical judgment. A higher minimum sensitivity than minimum specificity is chosen since false negative test results are much more clinically relevant for the patient than false positive test results, as the consequences of an undetected hemorrhage is more serious than those of a false alarm. In addition, a clinical examination will follow in any case to verify the test results.

The second primary global hypothesis considers the three-level traffic light decision system. Due to the hierarchical order of primary hypotheses, the second primary global hypothesis can only be evaluated in a confirmatory way if the first global primary hypothesis leads to a significant result. The hierarchical order of hypotheses requires no adjustment of the type I error rate.

The analysis of the first global primary hypothesis contains the estimation of the sensitivity and specificity of the IP using the defined cut-off value 12 mmHg. For the estimation, a mixed binary logistic regression model is used^31^: The binary test result represents the dependent variable, the true disease status is included as fixed effect. Additionally, a random-intercept is included for each center. Sensitivity is estimated via the calculation of the marginal mean for a diseased individual. False-positive rate, which is the counter probability of the specificity, is estimated via the calculation of the marginal mean for a non-diseased individual. A two-sided 95%-logit confidence interval for one proportion is calculated for sensitivity and specificity with p-values, respectively,^8^ since this type of confidence interval is range preserving.^32^ If the mixed binary logistic regression model does not converge, a non-parametric multi-factorial approach is used to estimate sensitivity and specificity.^33^ With this approach, separate estimates of sensitivity and specificity, respectively, are calculated for each study site and finally weighted averaged. Two-sided 95%-logit confidence intervals for the averaged sensitivity and specificity and p-values based on analysis of variance type statistics are reported.

The first primary null hypothesis can be rejected if the lower limits of both confidence intervals are above the according pre-defined minimum threshold (0.8 for sensitivity and 0.65 for specificity).^27^

The analysis of the third primary endpoint regarding the AUC of the three-level decision rule is performed in analogy to the analysis of the first co-primary pair. The according null hypothesis can be rejected if the lower limit of the confidence interval is above the according pre-defined minimum threshold of 0.7.

#### 3.4.2 Stage 2

All analyses will be performed in accordance with the intention-to-treat principle. All patients are analyzed as randomized. There are two primary hypotheses which are tested hierarchically. The first primary hypothesis aims to test for superiority regarding the “incidence proportion of alerting events within 48 h” in the intervention group compared to the SOC monitoring group. The second primary hypothesis aims to test for non-inferiority regarding the “composite endpoint of death of any cause, surgical interventions for suspected hemorrhage which did not show serious bleedings and serious adverse events” until one month of follow-up in the IP group compared to the SOC monitoring group. Based on clinical judgment, the clinically relevant non-inferiority margin is set to a hazard ratio of 1.2.

The first primary endpoint is the comparison between patients in the intervention group who have a technical alarm detected by the IP and/or a clinical alarm triggering surgical intervention due to clinical symptoms and patients in the control group who have a clinical alarm triggering surgical intervention due to clinical symptoms. A Bayesian mixed logistic regression model is calculated with the grouping variable (intervention vs. control) as factor and center as random effect, estimating finally an adjusted odds ratio and its corresponding two-sided 95% credible interval. An informative robust prior distribution based on stage I analysis is placed on the fixed intercept value. Standard weakly informative priors, for example, Cauchy and half-Cauchy distributions with appropriately large standard deviations^34^ are assigned to the remaining parameters. Sensitivity to prior choice will be investigated.

The seamless design presented here aims to incorporate the information available from stage 1 into the control group of the second stage in order to minimize the sample size, costs and recruitment time. Due to the presence of the additional—at this point external—controls from stage 1 the estimator in the randomized control group and in the additive control group are not treated equally.

Pocock^16^ provided criteria in order to evaluate the comparability of historical data with current trials data in cluster-randomized controlled trials (cRCTs). When deciding to use historical data in the analysis of a clinical trial, one should thus account for the possible heterogeneity between the trials. Hence, an evaluation of Pocock's criteria for the comparability of the additive data from stage 1 and the current control data of stage 2 is displayed in Table 1.

**Table 1.** Evaluation of Pocock's criteria.

Criteria by Pocock**^16^**	Application to HEDOS study
**(1)** “**The historical controls must have received a precisely defined standard treatment which must be the same as the treatment for the randomi**z**ed controls.**”	In stage 1, all patients are going to be treated according to routine clinical care, which is also intended to be applied in the control group of stage 2.
**(2)** “**The historical controls must have been part of a recent clinical study which contained the same requirements for patient eligibility.**”	Since this is a seamless design and the research questions refer to the same patient populations, the eligibility criteria are defined in the same way for both stages.
**(3)** “**The methods of treatment evaluation must be the same.**”	The primary endpoints of stage 2, that is, the incidence proportion of alerting events as well as the composite endpoint study as defined in Section 2.5.2, will be measured as secondary endpoints in stage 1, in the same manner as in stage 2. These endpoints will be analyzed descriptively in stage 1.
**(4)** “**The distributions of important patient characteristics in the additive controls should be comparable with those in the new trial.**”	An explorative analysis will be performed in order to check whether there will be any differences in baseline characteristics between the additive controls from stage 1 and current controls in stage 2.
**(5)** “**The previous studies must have been performed in the same organi**z**ation with largely the same clinical investigators.**”	The HEDOS study involves the seamless design. Both stages of the overall trial are planned, organized and carried out by the same clinical investigators from the beginning.
**(6)** “**There must be no other indications leading one to expect differing results between the randomi**z**ed and historical controls.**”	The overall quality of standard of care monitoring may have improved over time. To our knowledge, there are no other indications for substantial differences between the trials.

The study largely meets Pocock's criteria listed in Table 1. However, there is still some unknown bias resulting from the external controls and heterogeneity between the study data, which suggests some downweighting of the available external information.

The primary hypothesis in stage 2 will be analyzed using Bayesian inference.

The aim is to summarize the external information from stage 1 regarding the parameter(s) of interest by quantifying it with a suitable prior distribution, and update this information using the knowledge gathered during the current trial, that is, stage 2 study. In particular, we adopt a dynamic power prior method with a cap on the maximum amount of stage 1 information borrowed as quantified in terms of ESS^14,15^ The ESS corresponds to the amount of information contained in the prior distribution of stage 2 expressed as a hypothetical number of study sites, $ESS_{1}$ from stage 1. The choice of a suitable prior's ESS depends on how much external information should be used in statistical inference for stage 2. A threshold is fixed that reflects the maximum amount of hypothetical study sites that are incorporated into the prior with respect to the number of study sites who will be included in the current trial. As a large number of stage 1 study sites is expected compared to stage 2, the ESS is capped at 15, so $ESS_{1}=\;min(N_{1},15)$ , which corresponds to 30% of the expected number of study sites included in stage 1 $\;(N_{1}=50).$ This is a subjective choice and a reasonable compromise between the maximum number of study sites one would save in the recruitment in stage 2 (from sponsor's point of view) and the avoidance of potential bias caused by the use of the external information in case of prior-data conflict (from statistical point of view). It is usually accepted that $ESS_{1}<N_{2}$ in order to avoid the prior distribution dominates the actual data.^25^

The power prior method enables the robust incorporation of external data into the analysis of a current clinical trial by discounting the informativeness of the prior arising from such external data according to a parameter $a_{0}\in [0,1]$ , where 0 corresponds to no borrowing and 1 to full borrowing, to which the external data likelihood is raised (14,15); $a_{0}$ can be fixed a priori or dynamically estimated based on the observed heterogeneity between the current and external data source. Here we adopt a marginal version of the power prior combined with a dynamic approach, as in (33) and described in the following.

The posterior distribution for the parameter on which borrowing is desired is obtained from stage 1 data, under standard weakly or non-informative prior distributions. Such a posterior distribution is then approximated by a Gaussian distribution. Since it is expected that a much larger number of patients and study centers are available in stage 1 as compared to stage 2, stage 1 posterior—stage 2 prior—variance is inflated to avoid prior dominance on stage 2 inferences, as well as discounted based on observed bias.^35^ In particular, let θ denote the parameter on which borrowing is desired and $Var[\theta |D_{1}]$ its stage 1 marginal posterior variance with $D_{1}$ being the information from stage 1. Let $N_{1}$ be the number of stage 1 study centers available for information borrowing. Stage 2 prior variance is then obtained as $Var[\theta |D_{1}]\cdot \frac{N_{1}}{a_{0}ESS_{1}}$ , where $a_{0}\in [0,1]$ is the power prior parameter with $a_{0}=0$ corresponding to full discard of stage 1 information and $a_{0}=1$ to full incorporation (up to $ESS_{1})$ . The parameter $a_{0}$ is planned to be estimated adaptively from stages 1 and 2 observed data, according to the method presented by Ollier et al.^36^ and Calderazzo et al.^35^ In particular, the power prior parameter as described above is supplemented by a commensurability parameter, γ , using a measure of distribution distance, which measures a possible heterogeneity between the information from stages 1 and 2. When information is very heterogeneous, a non-informative prior should be preferable; when it is consistent, a complete borrowing is preferred.. The adaptive estimation of power parameter $a_{0}$ effectively robustifies the analysis against the effect of unexpected heterogeneity between stage 1 and stage 2 controls. Dynamic borrowing limits both mean squared error and Type I error rates inflations.^13^

Finally, a table with stage 2 posterior means and 95% credible intervals, as well as 25%, 50% and 75% quantiles will be reported.^37^ Further, a graphical presentation of the posterior distribution using, for example, univariate kernel density estimates or multivariate scatter plots will be provided.

The second primary endpoint will be compared between the ISAR-M-THYRO and the standard procedure group by using Cox proportional hazards model to estimate hazard ratios and corresponding two-sided 95% confidence intervals. Event probabilities at the different time points are reported based on Kaplan–Meier estimators. The second primary hypothesis will only be evaluated in a confirmatory sense if the first primary hypothesis leads to a significant test result.

## 4 Discussion and conclusion

We present a phase III/IV seamless design in diagnostic research which is the first study concept to be planned and implemented in the context of diagnostic research. The planning of the sample size of both stages and the implementation of the entire seamless design is complex and innovative from a statistical-methodological point of view.

So far, there is not much research concerning the usage adaptive designs in diagnostic studies compared to therapeutic trials.^3,8,9,38,39^ From regulatory point of view, Bothwell et al.^40^ did a literature review on adaptive designs in clinical trials and highlighted a slight trend on the reception of adaptive designs by regulatory agencies in the USA and Europe of adaptive designs. Further, the U.S. Food and Drug Administration provided guidance on how to plan and implement adaptive designs for clinical studies when used in medical device development programs^41^ as well as the development of drugs and biologics^42^ which can be used by sponsors and clinical researchers.

With this article, we want to emphasize the methodology as well as practical applicability of the design and make the reader and perhaps future users aware of the aspects that need to be considered when planning and evaluating this design. Hence, this work can serve as a template for other biostatisticians.

The medical device ISAR-M THYRO presented here is being investigated for the first time. Due to missing preliminary studies and uncertain assumptions in the initial sample size planning, the calculated sample sizes are rather insecure. The integration of the blinded design for sample size recalculation during the first stage provides us the opportunity to overcome this and gain initial insights into the study.

The second interim analysis to recalculate the sample size for the second stage will be performed after completion of the first stage. This will be unblinded since we obtain appropriate estimators for the diagnostic accuracy of the medical device as well as incidence proportion of alerts indicated by the IP.

A direct connection of the two study objectives in one single study by evaluating the diagnostic accuracy of the medical device on the one hand, for example, as an interim analysis, and the importance regarding a patient-relevant endpoint in the final analysis on the other hand on the same patient collective is not possible, since the treatment needs to be in accordance with the current health standards, including the current standard diagnostic setup. Therefore, we decided on a phase III/IV seamless design. At first, we aim for the certification and regulatory approval of the device. Subsequently, these patients will receive treatment according to the current standards anyway, allowing this group and study sites to be included in the control arm of the phase IV study, that is, this study is supplemented by external controls from the same clinical setup. Classical randomized test-treatment trials, as conducted in stage 2, generally require a high sample size. By re-using the data from the first stage, the overall sample size number of stage 2 centers can be reduced by the number of centers included in stage 1, $N_{1}\;=\;50$ , downweighted to 15 to avoid prior domination, as well as overall study duration can be shortened by the theoretical recruiting time required in stage 1. Hence, fewer patients are at risk in the cRCT (stage 2). Furthermore, the planned interim analyses allow us a more accurate sample size estimation for the cRCT based on valid observations by interim analysis of stage 1.

The integration of external data from stage 1 in stage 2 requires a careful planning of the methodological aspects. It is mandatory to define precisely the list of variables describing the inclusion and exclusion criteria, as well as possible confounding factors for the treatment effect, in order to investigate possible heterogeneity between the external and current trial.

The acceptance of additional controls into a randomized trial has already been discussed by several research groups.^13–16,43–45^ In the previous section, we have described criteria that can be used to evaluate the comparability of distribution of important patient characteristics as well as eligibility criteria and the clinical setup in both stages of the seamless design.

In stage 2, the aim is to evaluate the IP regarding patient-relevant outcomes. Thus, the subsequent management that these patients receive based on the IP results in the intervention group and based on the SOC monitoring in the control group, that is, revision surgery or not as part of the routine clinical care, is a key issue. In the first stage, all patients receive the treatment that corresponds to the current standards in routine care. Consequently, the treatment of the additional controls of stage 1 is the same as the treatment in the control group in stage 2 and will be evaluated in the same manner. Further, the entire seamless design is planned and performed by the same working group, including the sponsor, clinical investigators and biostatisticians. However, even if we assume that the study conditions are comparable in both stages, a serious potential of bias is still existing. Hence, only pooling of the additional controls with the randomized controls in stage 2 is not optimal. Adaptive down-weighting of the external information as described in 3.4.2, is an acceptable option in order to limit the bias under the worst possible scenario, while retaining efficiency gains when low heterogeneity is observed.

The immediate implementation of a phase IV after the phase III study gives us the opportunity to integrate an evaluation of the practical handling and usage of the IP. The daily clinical routine in the use of the device from the perspective of the patient as well as of the treating staff (nurses, physicians) will provide additional valuable information about the IP. This would correspond to a feasibility study in terms of practical functionality and acceptance by clinicians and patients. For this purpose, a randomly drawn sample (up to 100 patients) from the IP arm can be analyzed.

However, to ensure a streamlined process, all design and implementation aspects of the entire study process including the adaptive element must be planned carefully in advance, and as much specific guidance as possible must be provided to decision makers. This includes, above all, the blinding of the data (e.g., IP alert results) at the time of the interim analysis to the clinicians as well as to the sponsor. It is advisable to determine how the data collected in both phases will be combined for the final analysis and how the sample size will be calculated to meet the study objectives originally established for the two phases (separate studies). The planning of adaptive designs in the traditional sense for separately conducted studies requires prospective planning as well as pre-specification of the planned interim analyses and, if necessary, all rules for adaptation of the type I error rate. In adaptive seamless designs, this is also required by regulatory authorities.

The first step is the development and publication of a study protocol. In order to ensure the prospective character of the study, the protocol should outline the entire study process including both stages.^46^ In addition, a detailed statistical analysis plan for both stages of the seamless design in which all statistical aspects regarding study design, definitions, as well as interim and final analysis are predefined, should be in place before stage 1 study starts.

Furthermore, it is important to keep in mind here that the overarching objectives of phase III and phase IV studies differ. The success of phase III is linked to approval of adequate diagnostic accuracy of the IP. Before patient recruitment in phase IV is allowed to start, this needs to be ensured. In randomized trials, the treatment decision is based on the results of the diagnostic tests applied in order to demonstrate the relevance of the test in terms of a patient outcome. Indeed, this can only be achieved if the first stage has been successfully completed, thus, there may be a time lag in the study process.

Randomization is only performed in the randomized test-treatment study (stage 2). Since an enrichment by external controls from the first stage is performed in the second stage, the problem of potentially unbalanced randomization should be taken into account. To avoid prior domination, information equivalent to that of at most 15 stage 1 study centers will be included in the analysis, requiring further 10 stage 2 study centers to be randomized to control.

Control study sites that are recruited before the intervention arm in stage 2, that is, the external controls from stage 1, can also be referred to as non-concurrent controls and control study sites which are recruited at the same time with the study sites of the intervention arm as concurrent controls.^47^ The comparison of the intervention and control arm by simply pooling the concurrent with non-concurrent controls can be biased if there are time trends in the control data, for example, due to a modification of the SOP monitoring with time or a time delay due to CE certification of the IP or due to other external effects such as a pandemic or seasonal effects. This might lead to an impact on the type I error rate and marginal power.^48^ The modeling of time trends when using non-concurrent controls is particularly applied in platform trials, for example, by integrating interaction effects with group and time^47,48^ or applying weighted regression models.^48^ In an ideal setting, we would suppose that the distribution of controls in stage 1 matches the distribution of controls in stage 2. Hence, in Section 3.4.2, we applied Pocock's^16^ criteria to assess the degree of similarity between the two distributions. Even if we assume that the criteria are mostly satisfied, a potential bias caused by various factors over time cannot be excluded. Thus, as one possible workaround, we will adaptively estimate a factor $\alpha_{0}$ to downweight the influence of external controls in stage 2 based on observed heterogeneity (see 3.4.2). Moreover, if the randomization procedure as described in 3.1 is carried out, then the balance in patient characteristics and responses across the different stages might be maintained.

Before deciding to choose a seamless III/IV design for a project, one should consider whether these challenges will be a serious issue or not.

The sponsor of the study, health and regulatory authorities, ethics committees, the medical community and the patients are aiming for an efficient and successful process in the evaluation of medical products, such that it can be made available as early as possible to the patients. We are of the opinion that carefully planned and conducted studies based on seamless designs are an important tool which can help to fulfill these requirements.

In future work, statistical properties of the proposed design will be investigated in more detail in simulation studies.

## Trial status

The HEDOS study has been officially approved by the German Institute for Drugs and Medical Devices (Bundesinstitut für Arzneimittel und Medizinprodukte (BfArM)).

## Supplemental Material

sj-docx-1-smm-10.1177_09622802241227951 - Supplemental material for A diagnostic phase III/IV seamless design to investigate the diagnostic accuracy and clinical effectiveness using the example of HEDOS and HEDOS II Supplemental material, sj-docx-1-smm-10.1177_09622802241227951 for A diagnostic phase III/IV seamless design to investigate the diagnostic accuracy and clinical effectiveness using the example of HEDOS and HEDOS II by Amra Pepić, Maria Stark, Tim Friede, Annette Kopp-Schneider, Silvia Calderazzo, Maria Reichert, Michael Wolf, Ulrich Wirth, Stefan Schopf and Antonia Zapf in Statistical Methods in Medical Research

## Abbreviations

HEDOS: Thyroid HEmorrhage DetectOr Study
AUC: area under the curve
ROC: receiver operating characteristics curve
cRCT: cluster-randomized controlled trial
SOC: standard of care
IP: investigational product
$ESS_{1}$: effective sample size.
