# Supplementary material for: A diagnostic phase III/IV seamless design to investigate the diagnostic accuracy and clinical effectiveness using the example of HEDOS and HEDOS II
Source: Stat Methods Med Res. 2024 Feb 7;33(3):433–48. doi: 10.1177/09622802241227951 (PMC10981198; doi:10.1177/09622802241227951)
Supplement: sj-docx-1-smm-10.1177_09622802241227951 - Supplemental material for A diagnostic phase III/IV seamless design to investigate the diagnostic accuracy and clinical effectiveness using the example of HEDOS and HEDOS II [file sj-docx-1-smm-10.1177_09622802241227951.docx]

**A diagnostic phase III/IV seamless design to investigate the diagnostic accuracy and clinical effectiveness using the example of HEDOS and HEDOS II**

**Supplementary Material**

**Amra Pepic^1*^, Maria Stark^2^, Tim Friede^3^, Annette Kopp-Schneider^4^, Silvia Calderazzo^4^, Maria Ils^5^, Michael Wolf^5^, Ulrich Wirth^6^, Stefan Schopf^7^, Antonia Zapf^1^**

***Correspondence:** [a.pepic@uke.de](mailto:a.pepic@uke.de)

**^1^Institute of Medical Biometry and Epidemiology, University Medical Center Hamburg-Eppendorf, Christoph-Probst Weg 1, 20246 Hamburg, Germany.**

**Full list of author information is available at the end of the article.**

**Sample size calculation for stage 2**

**Derivation of assumptions**

For the first primary endpoint in stage 2, sample size calculation is performed in order to detect a difference in the incidence proportion of first clinical and technical events (red and yellow level) in the IP group, as defined in section 2.4.1, compared to the incidence proportion of clinical alerts in the control group (SOC monitoring).

For the ISAR-M detector we assume a sensitivity of 99% and a specificity of 95%. Assumptions regarding the sensitivity and specificity of the SOP monitoring are not available.

Overall, 1.5% of patients are expected to have symptomatic hemorrhage events, which is also assumed in the stage 1 study. We therefore assume that only this proportion at most will be detected in the control group. However, we assume that in the control group most patients who do not have hemorrhage events will be detected as green patients according to the three level traffic light system. Therefore, we assume that the SOC monitoring has a specificity of 99%.

If the detector has shown high diagnostic accuracy in the stage 1 study, we assume that it will serve as the gold standard in the stage 2 study.

Thus, the aim is to detect not only the symptomatic hemorrhage events in the IP group but also the asymptomatic hemorrhage events that are in the yellow group of the three level traffic light system.

If we now vary the prevalence of asymptomatic and symptomatic three level traffic light system events, we can calculate how high the sensitivity in the control group could be based on the defined specificity of the standard method. In the following, we assume that the total proportion of both symptomatic and asymptomatic hemorrhage events in the study is 7 %, as this is clinically relevant, then we suppose that of these, only the 1.5% symptomatic events are also detected by SOC monitoring. Thus, the sensitivity of the IP would be $\frac{0.015}{0.07} = 0.21$. In Table 1, different scenarios for the calculation of the sensitivity of SOC monitoring are listed.

Applying these assumptions, the calculation of the expected incidence proportion in both study arms follows Hot et al. 2021[9] and results in 2.4% for the control arm and 11.5% in the IP arm.

| Sensitivity  IP | Specificity  IP | Sensitivity SOC monitoring | Specificity SOC monitoring | prevalence | Expected event proportion  IP (red + yellow level) | | Expected event proportion SOC monitoring (red level) |
| --- | --- | --- | --- | --- | --- | --- | --- |
| 0.99 | 0.95 | 0.75 | 0.99 | 0.02 | 0.0688 | 0.0248 | |
| 0.99 | 0.95 | 0.50 | 0.99 | 0.03 | 0.0782 | 0.0247 | |
| 0.99 | 0.95 | 0.37 | 0.99 | 0.04 | 0.0876 | 0.0246 | |
| 0.99 | 0.95 | 0.30 | 0.99 | 0.05 | 0.0970 | 0.0245 | |
| 0.99 | 0.95 | 0.25 | 0.99 | 0.06 | 0.1064 | 0.0244 | |
| 0.99 | **0.95** | **0.21** | **0.99** | **0.07** | **0.1158** | **0.0243** | |
| 0.99 | 0.95 | 0.19 | 0.99 | 0.08 | 0.1252 | 0.0242 | |
| 0.99 | 0.95 | 0.17 | 0.99 | 0.9 | 0.1346 | 0.0241 | |
| 0.99 | 0.95 | 0.15 | 0.99 | 0.1 | 0.1440 | 0.0240 | |

*Table 1: Different scenarios of sensitivity and specificity of the IP and SOC monitoring in stage 2.*

**Two-sample proportion test for cluster-randomized trials**

In stage 2, a cluster-randomized trial is considered where the first primary hypothesis tests whether there is a difference regarding the incidence proportion of alerts in the intervention group (*Int*) compared to the control group (*Ctrl*). Hence, the sample size calculation involves a comparison of two binomial proportions $\theta_{Int}$ and $\theta_{Ctrl}$ based on two independent samples, respectively. Let $\alpha$ denote the type I error probability and $1-\beta$ the type II error probability. Accordingly, $z_{1-\alpha}$ and $z_{1-\beta}$ are the critical values of the standard normal distribution with upper tail probability of $1-\alpha$ and $1-\beta$, respectively. We are using a two-sided test to level $\alpha$ testing the hypotheses $H_{0}:\Delta=0$vs. $H_{1}:\Delta\neq0$, where $\Delta= \theta_{Int}- \theta_{Ctrl}.$ Therefore, in an individual-randomized trial, the sample size formula for testing the inequality of two binomial proportions $\theta_{Int}$ and $\theta_{Ctrl}$ with equal allocation of patients to the two study arms can be performed along the following lines[42]:

$$n= \frac{\left( z_{\frac{\alpha}{2}}+z_{\beta} \right)^{2}[\theta_{ctrl}\left( 1-\theta_{Ctrl} \right) +\theta_{Int}(1-\theta_{Int})]}{\Delta^{2}},$$

where $n$ is the calculated number of patients per group.

For a cluster-randomized trial, the intervention is randomized over $k$ clusters per arm each of size $m$, to provide a total of $N = m\cdot k$ individuals per arm.

Then, the number of cluster required per study group, is given by:

$$k= n\cdot(1+(m-1)\cdot ICC)$$

where $ICC$ is the Intraclass-Correlation Coefficient (ICC), which indicated how strongly individuals within clusters are related to each other .
